# Annotations

**Published:** 1907-09-28

**Authors:** 


### ANNOTATIONS.

### Glasgow Royal Infirmary.

The Fates in recent years do not seem to have dealt in an altogether kindly spirit with this institution. The re-building scheme, planned now more than ten years ago, was attended by quite a series of controversies, and has only recently been placed on lines calculated to lead to a satisfactory conclusion. Now, it appears that a conflict has arisen between the managers of the infirmary and the governors of St. Mungo's College, which is the picturesque title applied to the medical school of the infirmary. When the College was first incorporated, very large ambitions were entertained in connection with it. The hope was cultivated that it would become affiliated to the University, and that it and the Royal Infirmary would develop into a friendly rival of the University school and Western Infirmary. Events have not proved favourable to these ambitions. The endowment necessary for affiliation has never been raised, and the number of students has, for various reasons, steadily declined. In short, it has become evident that the cultivation of a complete medical curriculum and the development of a successful medical college in association with the Royal Infirmary must be abandoned as impossible. But this by no means implies that the Infirmary has no function as a centre for clinical and pathological study. On the contrary, it possesses facilities in these directions which are probably unsurpassed by any hospital in the country. What is wanted is that these opportunities shall be open to the medical students of the University. When this is attained both institutions will gain an enormous advantage. How such a result may be brought about is the business of the men on the spot. But it is obvious that either local patriotism or a capacity for affairs must be singularly defective if the largest hospital in the city cannot be brought within the sphere of operations of the University medical school. Were this effected, the Western and Royal Infirmaries would offer a conjoined clinical service unequalled at any other centre, and the educational opportunities presented by the University would gain both in efficiency and attractiveness. At the same time, the Royal Infirmary would gain all the benefits which are associated with the systematic presence of well-filled clinical classes.

### Advertisement in High Places.

The question of individual advertisement is one which at not infrequent intervals arouses strong feeling and vigorous denunciation in many professional organisations, and high authorities in the medical press are often invoked to hurl the editorial thunder at the heads of the daring offenders. Whether the stir and agitation thus arising are worth the time and energy spent is a question on which there may be differences of opinion, but, without doubt, condemnation, if it is applied at all, ought to be applied on impartial principles. An advertisement which has recently appeared on the front sheet of the leading lay journal affords an opportunity of judging whether this claim is to receive practical recognition. The advertisement in question refers to a metropolitan hospital medical school, and, in addition to the usual statements about "special features" and "complete training," it includes a full list of the teaching staff, each professor and lecturer being named in connection with the subject he professes, and having duly set forth his academic and professional distinctions. In this list, Fellowships of the Royal College of Physicians and of the Royal College of Surgeons occupy a prominent position, and there are at least two names which may be found in exalted positions in these august organisations. We are not just now venturing to say that such an advertisement is unethical or improper. Our purpose is rather to record our inability to recognise any distinction between it and others which every now and then come under orthodox professional censure. The publication in a lay paper of the names of the staff of the local hospital or dispensary has been condemned again and again. Recently a similar sentence was directed against a large non-metropolitan medical school. It will be of interest to note whether equal denunciation will extend to the incident related in this note.

### The Royal Society of Medicine.

Now that the greater difficulties attending the amalgamation of the London medical societies and the establishment of the Royal Society of Medicine have been surmounted, it may be confidently anticipated that the serious work of the new Society will at once be entered on. Doubtless there will be minor troubles associated with organisation and administration, but these will disappear under the influence of experience and goodwill. Two questions of this order have already come to the surface. One relates to the extent of the privileges of the Fellows of the new society in regard to the various sections and meetings. Of course, the Fellows will enjoy the right to attend all meetings, whether general or sectional. But they will only be competent to take part in the business of those sections of which they are members, and membership means election by the section concerned excepting only those persons who come into one or more sections as belonging to a society which has taken part in the amalgamation, these being without any formality original members of the corresponding sections. At first sight it seems somewhat anomalous that a Fellow of the Society should be excluded from the business of any of the departments of the Society. But a little consideration will show that practice renders this restraint essential. It is imperative that each section should have appropriate representation on the Central Council, and this position might readily be put in peril were any section open to the interposition of a number of Fellows whose interest in the special work of such section was an indirect and remote one. Another anxiety in relation to the new Society is the manner in which Fellows and members of sections shall describe themselves in the Medical Directory. The short way, and we believe the best way, would be for the proprietors to refuse all titles relating to professional societies. Such announcements are of no substantial value, and they help to render burdensome a valuable, indeed an essential, volume.

